# Concurrent Multi-Beam Digital Predistortion Using FFT Beamforming and Virtual Arrays

**DOI:** 10.3390/s26082400

**Published:** 2026-04-14

**Authors:** Björn Langborn, Christian Fager, Rui Hou, Thomas Eriksson

**Affiliations:** 1 Department of Electrical Engineering, Chalmers University of Technology, 412 96 Göteborg, Sweden; langborn@chalmers.se; 2 Department of Microtechnology and Nanoscience, Chalmers University of Technology, 412 96 Göteborg, Sweden; christian.fager@chalmers.se (C.F.); rui.hou@ericsson.com (R.H.); 3 Ericsson AB, 164 83 Stockholm, Sweden

**Keywords:** digital predistortion, virtual array, power amplifier, MIMO, multi-beam, beam domain, precoding, FFT beamforming

## Abstract

A digital predistortion (DPD) scheme for concurrent multi-beam transmission in fully digital multiple-input, multiple-output (MIMO) systems, using Fast Fourier Transform (FFT) beamforming and so-called virtual-array processing, is proposed. In a MIMO array with nonlinear power amplifiers (PAs), transmitting multiple beams concurrently yields intermodulation products that end up in both user and non-user directions. In the setting with few users in a large array, the array dimension will typically be much larger than the number of generated intermodulation products. At the same time, linearization per PA is excessively costly for large arrays. This work shows that it is instead possible to linearize the system by producing predistorted user beams, and non-user intermodulation products, through DPD processing in a virtual array of a much smaller dimension than the physical array. Theoretical derivations and simulation examples show how this approach can lead to manyfold reductions in DPD complexity.

## 1. Introduction

Multi-antenna transmitters offer the possibility to spatially direct multiple beams (The concept of a beam in this paper will sometimes be used interchangeably with a precoded data stream, generalizing statements from line-of-sight to other channel conditions.) concurrently, which is of interest for many applications. For communication, it allows base stations to serve multiple users in different locations concurrently. For localization and sensing, utilizing multi-beam schemes can improve localization accuracy [1]. To utilize these possibilities fully in future radio systems, the number of transmit branches is expected to increase dramatically. Simultaneously, operating PAs with as high efficiency as possible always comes with a compromise in their linearity. However, generating distortion due to nonlinear operation is problematic for multiple reasons. Firstly, distortion will be emitted in-band in the directions of the users, deteriorating communication link quality. Secondly, distortion will be generated both in-band and out-of-band into non-user directions and can interfere with other systems. As such, nonlinear distortion must be considered to fulfill transmission requirements. To this end, DPD is widely adopted.

Numerous approaches have been proposed for linearizing multi-antenna transmitters, as reviewed in Section 1.1. In this work, we focus on systems employing fully digital beamforming. Prior contributions have targeted either distortion cancellation specifically in the user directions [2,3] or full-angle distortion suppression when a single dominant beam is transmitted [4]. Other recent state-of-the-art research has extended these ideas to scenarios with multiple simultaneous beams through so-called beam-domain DPD (BD-DPD) [5,6].

BD-DPD for multi-beam linearization is similar to multi-dimensional DPDs from the concurrent multi-band setting, such as 2D and 3D-DPD [7,8,9,10]. These linearization approaches take multiple user data streams as input to a DPD, and combine the streams to generate a set of multi-dimensional basis functions. As such, they suffer the drawback of a drastically increasing number of multi-dimensional basis functions as the number of user streams increases. One alternative approach to this in the multi-band setting is frequency relocation [11], which involves relocating bands to lie closer in frequency, and generating all the necessary basis functions for linearization through a single nonlinearity before inverse frequency relocation. No similar technique to this exists in the multi-beam setting, to the authors’ best knowledge.

### 1.1. Previous Work

Handling distortion emitted from multi-input, multi-output (MIMO) transmitters can be done differently depending on system requirements, available resources, and number of users to be served simultaneously. This subsection provides a brief overview of two main categories of prior work: distortion suppression through beamforming strategies and distortion mitigation through linearization.

A first line of work addresses nonlinear distortion by shaping the beamforming pattern such that distortion is nulled in specific directions, as demonstrated in [12]. In [12], a single-user scenario is studied, with reference to [13] that intermodulation will tend towards being radiated isotropically once multiple beams, or many channel taps, are considered. This glosses over the case when, e.g., there are multiple, but few, beams in relation to the array size. Additionally, a nullforming approach can prevent distortion from being transmitted in specific directions, but it will not be able to prevent distortion from being generated at the PA outputs and subsequently transmitted in many directions. For that, distortion compensation is needed.

A second approach to handling nonlinear distortion is thereby through linearization. In a fully digital beamforming MIMO system, the most straightforward approach to linearization is adopting one DPD per PA in the transmitter chain. While effective, this solution becomes costly in hardware, energy consumption, and computational complexity as the number of transmit branches grows. Consequently, several alternative linearization strategies have been proposed.

For a MIMO transmitter with a single beam, so-called full-angle DPD [4] has been proposed. Here, instead of linearizing *L* PAs with *L* equal-size DPDs, it is proposed to use a common DPD prior to the precoder, and to perform only minor tuning in the branches to consider branch-wise variations. It is shown to perform well, and to be able to effectively cancel distortion from the transmitter. It is worth noting that this approach still requires tuning for all the branches but one, which is used as reference. From their results, it can be seen that the tuning boxes are not insignificant in size, as their common DPD had 21 coefficients whilst the tuning boxes each had 3–10 coefficients. Furthermore, the DPD scheme considers only a single beam, while in practice it is of great interest to transmit multiple beams concurrently.

Several works have addressed multi-beam linearization. In [3], the goal is to cancel inter-user distortion arising from concurrent multi-beam transmission, and the approach is validated for two users. A related technique for three concurrent users is presented in [14]. However, neither of these studies considers distortion radiated in non-user directions.

In [5], BD-DPD was adopted, applying linearization prior to the beamformer and operating on user data streams rather than on per-antenna signals. With this approach, the DPD complexity scales in relation to the number of users, *K*, and user stream intermodulation products, rather than the number of antennas, *L*. For systems where K≪L, this can yield large complexity savings. However, as in 2D- and 3D-DPD for multi-band signals, the number of intermodulation products grows rapidly with *K*, leading to multi-dimensional basis functions such as $x_{1}x_{2}x_{3}^{*}$. This is significant for practical implementations that rely on lookup tables (LUTs) to reduce arithmetic computations: multi-dimensional basis functions require multi-dimensional LUTs, increasing both memory usage and interpolation/extrapolation complexity, as discussed in [15]. Lastly, BD-DPD assumes equal PAs across branches. In the presence of PA variations, the performance of BD-DPD is expected to deteriorate.

Other recent contributions include neural network-based DPD architectures applied prior to the precoder [16], as well as DPD strategies for hybrid beamforming systems [2]. Although the neural network approach in [16] obtains good performance and is supported by experimental validation, it does not offer new theoretical or architectural insights. Furthermore, it incurs high memory requirements and substantial floating-point computational costs. As for hybrid beamforming architectures, they are highly relevant to study. However, subarray-based approaches suffer reduced beamforming gain, whereas fully connected architectures entail significant complexity due to crosstalk effects that must be incorporated into the DPD unless model-order reductions are applied [2,17].

### 1.2. Key Contributions and Paper Organization

In this paper, a novel approach to DPD processing using a so-called virtual array is proposed for concurrent multi-beam linearization. The core idea is to process the user data streams through a low-dimensional virtual transmit array, and a corresponding virtual channel representation, whose operation reflects that of the actual array. In the physical transmitter, the *L* branches with nonlinear PAs generate intermodulation (IM) distortion that is radiated over the air (OTA) in both user and non-user directions. In the virtual array, by contrast, $L_{v}$ nonlinear DPD branches seek to generate the IM terms required to cancel the distortion from the physical array—while using far fewer branches, with $L_{v}\ll L$. This concept has similarities to how previous work performed dimensionality reduction for the multi-band setting, utilizing frequency domain sparseness, via frequency relocation [11] or non-uniform downsampling [18]. However, this work performs dimensionality reduction for the multi-beam setting by utilizing so-called beam-domain sparseness.

The specific contributions of this paper are as follows:

- A novel approach to multi-beam DPD for MIMO transmitters performing DPD in a small virtual antenna space is proposed and exemplified. It is shown how such an approach can lead to manyfold reductions in complexity.
- Practical considerations in implementing the proposed scheme are accounted for through simulation and discussion, considering multi-user cases, different channels, and lookup table (LUT) realizations.
- Simulation results verify the theoretical findings, show the applicability of the proposed scheme, and demonstrate how the DPD scales in computational complexity in relation to the number of users.

This paper is organized as follows: The assumed system model description is expanded upon in Section 2. Firstly, in Section 2.1, the model description is expanded upon in order to comprehend how distortion is spatially distributed in the beam domain during concurrent multi-beam operation. After that, in Section 2.2, Fast Fourier Transform (FFT) beamforming is described. This is followed in Section 3 by an example where the orthogonality of FFT beamforming highlights the sparsity of the beam domain after over-the-air (OTA) transmission. Section 4 goes on to describe how processing in a virtual array, smaller than the actual physical one, can produce the same linearization signals as per-PA (PP) DPD but at a lower computational cost. Lastly, numerical simulation results are presented in Section 5 to verify the proposed approach, alongside a conclusion in Section 6.

## 2. System Modeling

The system modeling of this work focuses on a discrete time-domain, baseband description of concurrent multi-beam transmission in a fully digital multi-branch transmitter setting, firstly by detailing how intermodulation products arising from nonlinear PAs can be understood in such a description, and then relating them to FFT beamforming to demonstrate the suitability of the proposed approach.

**Figure 1:**
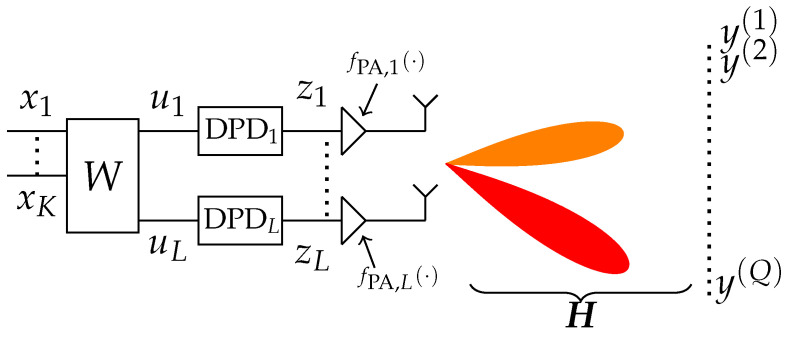
Concurrent multi-beam transmitter schematic of *K* user data streams $x_{1},\ldots ,x_{K}$, beamformed by W to constitute signals $u_{1},\ldots ,u_{L}$ that are passed through DPDs, transmitted through PAs on *L* antenna branches and observed in *Q* directions. Signals $z_{1},\ldots ,z_{L}$ are predistorted streams, $f_{\mathrm{PA},l}$ denote a nonlinear PA on branch *l*, H represents the wireless channel, and $y^{\left(q\right)}$ refers to the observed signal in direction (q).

Two key concepts used throughout this work are the beam domain and the antenna space. The beam domain refers to the domain in which signals have already propagated through a channel and formed beams as a result of beamforming. In Figure 1, this is illustrated by the colored beams (red and orange), which are observed over the air in various directions as signals $y^{\left(1\right)},\ldots ,y^{\left(Q\right)}$. Some beams carry the user streams $x_{1},\ldots ,x_{K}$, while others contain only IM distortion. In contrast, the antenna space refers to the signals on the different transmit branches, prior to channel propagation. In Figure 1, the antenna space encompasses the signals $u_{1},\ldots ,u_{L}$, $z_{1},\ldots ,z_{L}$ and also $f_{\mathrm{PA},1}\left(z_{1}\right),\ldots ,f_{\mathrm{PA},L}\left(z_{L}\right)$. The beam domain and antenna space are related through multiplication with a channel or beamforming matrix. For a channel $H\in C^{Q\times L}$, a signal $z\in C^{L\times N}$ in the antenna space, where *N* is the number of baseband data samples, the corresponding beam-domain signal is $Y=Hz\in C^{Q\times N}$. This relation is of particular interest when Q=L, and H is an orthogonal matrix, as will be later seen in the subsection on Fast Fourier Transform beamforming. Additionally, whilst this paper initially expands upon the relation between the antenna space and beam domain in the context of line-of-sight (LOS) channels, it is later addressed for non-LOS conditions as well.

To limit the scope of the paper, all signals $x_{1},\ldots ,x_{K}$ listed in this work are considered to be single-band signals. This can however be generalized to multi-band signals as well, with appropriate modification to the involved DPD units.

As a final comment, since this work considers fully digital beamforming, the numbers of antennas, transmit branches, and PAs are all equal and denoted by *L*.

### 2.1. Concurrent Transmission of Multiple Beams

Consider the schematic setup of Figure 1, but start by letting the DPDs be bypassed such that $z_{l}=u_{l}$, l=1,…,L. In this setup, the beamformer W is formed to direct user streams $x_{1},\ldots ,x_{K}$ to directions 1,…,Q in matrix form as

(1) $$W=\left[\begin{array}{ccc} w^{\left(1\right)} & \ldots & w^{\left(K\right)} \end{array}\right]\;,$$

with $\left|\right|w^{\left(k\right)}{\left|\right|}_{2}=1$, yielding PA input signals

(2) $$\left[\begin{array}{c} z_{1}\\ z_{2}\\ \vdots \\ z_{L} \end{array}\right]=\left[w^{\left(1\right)},\ldots ,w^{\left(K\right)}\right]\left[\begin{array}{c} x_{1}\\ x_{2}\\ \vdots \\ x_{K} \end{array}\right]\;.$$

Alternatively, the signal $z_{l}$ passed to PA *l* can be written as

(3) $$z_{l}=w_{l}^{\left(1\right)}x_{1}+\ldots +w_{l}^{\left(K\right)}x_{K}\;.$$

Now, it is known that Volterra series are well-suited to model a broad range of nonlinear functions, and as such, it is commonly applied to model saturation, soft clipping, and other effects in PAs. For practical application, different finite truncations focused on the lower-order nonlinear terms of the Volterra series are frequently used. This can be justified by the fact that lower-order nonlinear terms typically dominate the induced distortion. For qualitative analysis here, consider simply a third-order polynomial baseband PA model as

(4) $$\begin{array}{cc} f_{\mathrm{PA},l} & \left(z_{l}\right)=c_{1,l}z_{l}+c_{3,l}z_{l}{\left|z_{l}\right|}^{2}\;. \end{array}$$

where $f_{\mathrm{PA},l}$ refers to the nonlinear function of the PA on branch *l*, and $c_{1,l},c_{3,l}$ are modeling coefficients. Then, inserting (3) into (4) yields

(5) $$\begin{array}{cc} f_{\mathrm{PA},l}\left(z_{l}\right)=c_{1,l} & (w_{l}^{\left(1\right)}x_{1}+\ldots +w_{l}^{\left(K\right)}x_{K})+\\ c_{3,l} & \left(w_{l}^{\left(1\right)}x_{1}+\ldots +w_{l}^{\left(K\right)}x_{K}\right){\left|w_{l}^{\left(1\right)}x_{1}+\ldots +w_{l}^{\left(K\right)}x_{K}\right|}^{2}\\ =c_{1,l} & (w_{l}^{\left(1\right)}x_{1}+\ldots +w_{l}^{\left(K\right)}x_{K})+\\ c_{3,l} & (w_{l}^{\left(1\right)}w_{l}^{\left(1\right)}w_{l}^{(1)*}x_{1}|x_{1}{|}^{2}+\\ \; & w_{l}^{\left(1\right)}w_{l}^{\left(2\right)}w_{l}^{(2)*}x_{1}{\left|x_{2}\right|}^{2}+\\ \; & w_{l}^{\left(1\right)}w_{l}^{\left(1\right)}w_{l}^{(2)*}x_{1}^{2}x_{2}^{*}+\ldots )\;. \end{array}$$

First, assume that all PAs are equal, such that $c_{1,l}$ and $c_{3,l}$ are the same for each branch *l*. Then, let each PA output propagate through a channel vector $h_{l}=\left[\begin{array}{ccc} h_{l}^{\left(1\right)} & \ldots & h_{l}^{\left(Q\right)} \end{array}\right]$ from branch *l* to OTA location *q*, as 

(6) $$y^{\left(q\right)}=\left[\begin{array}{ccc} h_{1}^{\left(q\right)} & \ldots & h_{L}^{\left(q\right)} \end{array}\right]{\left[\begin{array}{ccc} f_{\mathrm{PA},1}\left(z_{1}\right) & \ldots & f_{\mathrm{PA},L}\left(z_{L}\right) \end{array}\right]}^{T}\;.$$

Consider the first-order linear terms

(7) $$\begin{array}{cc} y_{1\mathrm{st}}^{\left(q\right)} & =h_{1}^{\left(q\right)}c_{1}\left(w_{1}^{\left(1\right)}x_{1}+\ldots +w_{1}^{\left(K\right)}x_{K}\right)+\ldots \\ \; & +h_{L}^{\left(q\right)}c_{1}\left(w_{L}^{\left(1\right)}x_{1}+\ldots +w_{L}^{\left(K\right)}x_{K}\right)\;. \end{array}$$

Then it is known from beamforming theory that if $x_{1}$ is to go in direction (q), then beamforming weights can be chosen such that $w_{l}^{\left(1\right)}={\left(h_{l}^{\left(q\right)}\right)}^{*}$. This yields constructive interference $c_{1}\sum h_{l}^{\left(q\right)}w_{l}^{\left(1\right)}=c_{1}$. Then, by studying the third-order terms in (5), it can be seen that since $w_{l}^{\left(1\right)}w_{l}^{(1)*},w_{l}^{\left(2\right)}w_{l}^{(2)*}$ will be real constants, $x_{1}{\left|x_{1}\right|}^{2}$ and $x_{1}{\left|x_{2}\right|}^{2}$ will also go in direction (q). However, the summation of the IM products last listed in (5), $c_{3}\sum_{l}h_{l}^{\left(q\right)}w_{l}^{\left(1\right)}w_{l}^{\left(1\right)}w_{l}^{(2)*}$, typically has constructive interference in a direction other than (q). Generally, it can be seen that the terms of the form $w^{\left(i\right)}w^{\left(j\right)}w^{(k)*}$, where k≠i,j, will give rise to new beam directions of constructive interference, which are referred to as off-beams.

Two interesting observations can be made from (5), still assuming $c_{3,l}=c_{3}\;\forall l$. Firstly, the generated off-beam terms and directions can be deterministically computed from the intended transmit directions. To provide an example, off-beam terms for a static nonlinearity of order P=3 and for K=2,3,4 concurrent users are listed in Table 1. Secondly, the nonlinear products of the input streams are of a different form in the off-beams than for the in-beam. Thus, one needs to be aware of all beam directions and manage to generate the correct IM terms in the respective directions in order to linearize the system. This can readily be understood to grow more complex with more beams.

Simplified assumptions have been made in this subsection for qualitative analysis. The simple beamformer in (1) will later be further elaborated on in Section 4.3. Furthermore, the validity of the proposed method when PAs vary on different branches is investigated by simulation in Section 5.5, addressing the assumption of equal PAs. Lastly, it is well-known that PAs will often exhibit higher-order nonlinear behavior than order 3, as in (4), and that memory effects will be present unless the signal bandwidth is very narrow. Section 4.4 comments on introducing higher-order terms in the scheme, and whilst the analysis here was done for static PAs, memory can be added without any loss of generality.

### 2.2. Fast Fourier Transform Beamforming

FFT beamforming is one common way to efficiently limit the beamforming matrix to an orthogonal set of beam directions, by first defining

(8) $$\omega =e^{-j2\pi /L}\;.$$

Then, a one-dimensional discrete Fourier transform (DFT) beamformer matrix can be formulated as

(9) $$W_{\mathrm{DFT}}=\frac{1}{\sqrt{L}}\left[\begin{array}{cccc} 1 & 1 & 1 & \ldots \\ 1 & \omega & \omega^{2} & \ldots \\ 1 & \omega^{2} & \omega^{4} & \ldots \\ \vdots & \vdots & \vdots & \vdots \end{array}\right]=\frac{1}{\sqrt{L}}\left[\begin{array}{cccc} \omega^{\left(b_{1}\right)} & \omega^{\left(b_{2}\right)} & \omega^{\left(b_{3}\right)} & \ldots \end{array}\right]\;,$$

where $b_{i}\in \left[1,L\right]$ are beam indices indexing the beams on the DFT beamgrid. In implementation though, no matrix multiplication of $W_{\mathrm{DFT}}$ with data is needed, as would otherwise be indicated by (2). Instead, FFT algorithms can perform this computation at lower complexity.

It is worth noting that when the main beams are limited to a DFT beamgrid, $b_{i}\in \left[1,L\right]$, the off-beam directions will be restricted to the same beamgrid as well, given equal PAs. Furthermore, extension to two-dimensional beamforming is straightforward, by implementing FFT along both horizontal and vertical directions, but it is omitted from the derivations for brevity. All the analysis following in this paper can then be applied to each dimension, horizontal or vertical, separately.

With the system model established, the following section details how per-PA (PP) DPD produces predistorted streams and how intermodulation beam placement is affected by aliasing due to the antenna array being finite and discrete.

## 3. Per-PA DPD

This section details the processing steps for transmitting *K* user streams using per-PA (PP) DPD, as illustrated in Figure 1. A three-user example is used to demonstrate two important aspects: (i) the inherent sparsity of the resulting beam-domain representation for large *L* and (ii) an effect referred to as array aliasing, which appears due to the finite and discrete nature of the antenna array.

### 3.1. Per-PA DPD Example for Three Concurrent Users

Let the antenna array be a linear, one-dimensional array in space, and restrict the beamformer to a DFT beamgrid. As such, this subsection follows the notation of Figure 1, in which $x_{1},\ldots ,x_{K}$ represent user streams and *L* represents the number of transmit branches, whilst the beamformer W is represented by $W_{\mathrm{DFT}}$ from (9).

Consider that there are L=16 transmit branches (While L=16 is chosen here for clarity of exposition, practical systems would typically employ significantly larger arrays), transmitting $x_{1},x_{2},x_{3}$ to K=3 users in different LOS directions. Being restricted to a DFT beamgrid, the users can be located at beam indices $\left\{b_{1},b_{2},b_{3}\right\}\in \left[1,L\right]$. To exemplify, let $b_{1}=1,b_{2}=2,b_{3}=5$ such that

(10) $$\begin{array}{cc} z=W_{\mathrm{DFT}}x & =\frac{1}{\sqrt{L}}\left[\begin{array}{cccc} 1 & 1 & 1 & \ldots \\ 1 & \omega & \omega^{2} & \ldots \\ 1 & \omega^{2} & \omega^{4} & \ldots \\ \vdots & \vdots & \vdots & \vdots \end{array}\right]\left[\begin{array}{c} x_{1}\\ x_{2}\\ 0\\ 0\\ x_{3}\\ 0\\ \vdots \end{array}\right]\\ \; & =\frac{1}{\sqrt{L}}\left[\begin{array}{c} x_{1}+x_{2}+x_{3}\\ x_{1}+\omega x_{2}+\omega^{4}x_{3}\\ x_{1}+\omega^{2}x_{2}+\omega^{8}x_{3}\\ \vdots \end{array}\right]\\ \; & =\omega^{\left(b_{1}\right)}x_{1}+\omega^{\left(b_{2}\right)}x_{2}+\omega^{\left(b_{3}\right)}x_{3}\;. \end{array}$$

Assume for analytical brevity that each DPD is represented by the same nonlinear function f(·). Applying the nonlinear DPDs to each of these beamformed data streams, with a third-order nonlinearity $f\left(x\right)=\theta_{1}x+\theta_{3}x{\left|x\right|}^{2}$ as the DPD function, then

(11) $$\begin{array}{cc} \; & f(W_{\mathrm{DFT}}x)=\\ \; & \underset{L\times 1}{\underset{︸}{\frac{1}{\sqrt{L}}\left[\begin{array}{c} \theta_{1}\left(x_{1}+x_{2}+x_{3}\right)+\frac{1}{L}\theta_{3}\left(x_{1}+x_{2}+x_{3}\right){\left|x_{1}+x_{2}+x_{3}\right|}^{2}\\ \theta_{1}\left(x_{1}+\omega x_{2}+\omega^{4}x_{3}\right)+\frac{1}{L}\theta_{3}\left(x_{1}+\omega x_{2}+\omega^{4}x_{3}\right){\left|x_{1}+\omega x_{2}+\omega^{4}x_{3}\right|}^{2}\\ \theta_{1}\left(x_{1}+\omega^{2}x_{2}+\omega^{8}x_{3}\right)+\frac{1}{L}\theta_{3}\left(x_{1}+\omega^{2}x_{2}+\omega^{8}x_{3}\right){\left|x_{1}+\omega^{2}x_{2}+\omega^{8}x_{3}\right|}^{2}\\ \vdots \end{array}\right]}}\;, \end{array}$$

is obtained. How these signals will be spatially distributed OTA can be seen by transformation to the beam domain, achieved through an inverse Fourier transform. The user streams will then be clearly separated in the beam domain, like the beams in Figure 1. With the matrix $W_{\mathrm{IDFT}}=LW_{\mathrm{DFT}}^{*}$, where * denotes the Hermitian transpose, factors *L* outside the matrix cancel out, resulting in data streams

(12) $$\begin{array}{c} LW_{\mathrm{DFT}}^{*}f\left(W_{\mathrm{DFT}}x\right)=\left[\begin{array}{c} \theta_{1}x_{1}+\frac{1}{L}\theta_{3}x_{1}\left(\right|x_{1}{|}^{2}+|x_{2}{|}^{2}+|x_{3}{|}^{2})\\ \theta_{1}x_{2}+\frac{1}{L}\theta_{3}x_{2}\left(\right|x_{1}{|}^{2}+|x_{2}{|}^{2}+|x_{3}{|}^{2})\\ \frac{1}{L}\theta_{3}x_{2}^{2}x_{1}^{*}\\ \frac{1}{L}\theta_{3}x_{1}x_{3}x_{2}^{*}\\ \theta_{1}x_{3}+\frac{1}{L}\theta_{3}x_{3}\left(\right|x_{1}{|}^{2}+|x_{2}{|}^{2}+|x_{3}{|}^{2})\\ \frac{1}{L}\theta_{3}x_{2}x_{3}x_{1}^{*}\\ 0\\ \frac{1}{L}\theta_{3}x_{3}^{2}x_{2}^{*}\\ \frac{1}{L}\theta_{3}x_{3}^{2}x_{1}^{*}\\ 0\\ 0\\ 0\\ \frac{1}{L}\theta_{3}x_{1}^{2}x_{3}^{*}\\ \frac{1}{L}\theta_{3}x_{1}x_{2}x_{3}^{*}\\ \frac{1}{L}\theta_{3}x_{2}^{2}x_{3}^{*}\\ \frac{1}{L}\theta_{3}x_{1}^{2}x_{2}^{*} \end{array}\right]\;. \end{array}$$

Each entry at a separate index here represents a signal going in a unique beam direction. Two observations are worth noting. The first observation is that only 12 out of the 16 entries are non-zero. This is unsurprising, but important, as Table 1 shows that there can only be up to nine off-beam terms going in potentially different directions, in addition to the K=3 user directions, for a third-order nonlinearity. This is also true when *L* is a very large value. Thus, for large *L*, there are very few low-order signal components in relation to the array size. As for the second observation, concerning where each signal component ends up on the beamgrid, it is detailed in the following subsection.

### 3.2. Array Aliasing

In (12), each intermodulation (IM) term maps to a beam index determined by adding or subtracting the corresponding precoder beam indices. For example, the intermodulation term ${\left(\omega^{\left(b_{2}\right)}x_{2}\right)}^{2}{\left(\omega^{\left(b_{1}\right)}x_{1}\right)}^{*}=\left(\omega^{\left(b_{2}\right)}\omega^{\left(b_{2}\right)}\omega^{(b_{1})*}\right)x_{2}^{2}x_{1}^{*}$ indeed produces the intermodulation term $x_{2}^{2}x_{1}^{*}$ up on beam index $b_{2}+b_{2}-b_{1}=2+2-1=3$. However, this simple index addition does not always yield a valid beam index in the range [1,L]. For instance, the IM product ${\left(\omega^{\left(b_{1}\right)}x_{1}\right)}^{2}{\left(\omega^{\left(b_{2}\right)}x_{2}\right)}^{*}$ corresponds to the index $2b_{1}-b_{2}$, which would fall outside [1,L]. Instead, this term is aliased back in to the beamgrid range [1,L] according to

(13) $$\begin{array}{cc} \bar{b_{i_{1}}+b_{i_{2}}-b_{i_{3}}}=b_{i_{1}}+ & b_{i_{2}}-b_{i_{3}}-\\ \; & L\lfloor \frac{b_{i_{1}}+b_{i_{2}}-b_{i_{3}}-1/2}{L}\rfloor \;, \end{array}$$

for $\left\{b_{i_{1}},b_{i_{2}},b_{i_{3}}\right\}\in \left[1,L\right]$, $\left\{i_{1},i_{2},i_{3}\right\}\in \left[1,\ldots ,K\right]$, and where the bar notation represents the aliasing operation. With this in mind, it can be noted that whilst the example of Section 3.1 produced 12 uniquely placed data streams of main beam and intermodulation beam data, aliasing can, for a different beam configuration $\left\{b_{1},b_{2},b_{3}\right\}$, end up overlapping intermodulation terms to go in the same beam directions. This will lead to distortion being concentrated in fewer beam directions.

## 4. Virtual-Array DPD

**Figure 2:**
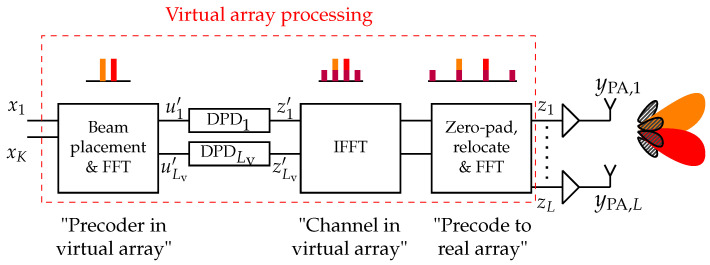
Schematic of DPD using virtual-array processing. The bar plots exemplify the beam space representation of beamforming the data streams to beams and induced IM beams. Red and orange correspond to desired user signals, while purple corresponds to the injected distortion by the DPDs to cancel out the unwanted distortion, shown in striped dark gray. Here, the number of virtual branches $L_{v}$ can always be made less than or equal to the number of antenna branches *L*.

In this section, virtual-array DPD (VA-DPD) processing, as shown in Figure 2, is proposed to reduce the complexity compared to applying a DPD per PA. As previously described in Section 1.2, the VA-DPD aims to produce predistorted user streams and off-beam IM terms in a low-dimensional virtual array, and use these to linearize the larger, real transmit array. This processing conceptually follows four main steps. First, user streams are precoded to the antenna space of a virtual array, by placement on a beamgrid and through an FFT. The beam placement in the virtual array depends on the precoding to be done in the real array, which will be detailed later. Second, nonlinear DPDs are applied to the data on each of the $L_{v}$ virtual antenna space branches. Third, an IFFT is performed, acting as a virtual channel and to separate user streams and off-beam IM terms in the virtual beam space. Fourth, and last, the predistorted streams that have been generated are precoded to the real array. The mapping in the virtual domain is chosen such that the predistorted terms generated by the virtual array match those needed to cancel the distortion produced in the physical array. In the following subsections, the derivations of Section 3.1 will be used as a starting point to give mathematical motivation as to how a smaller virtual array, in place of the original array, can be used for performing DPD. This is presented first with an example comparison to the per-PA case, followed by presenting special beam configuration cases in which the complexity can be reduced even further. Finally, generalizations to more general channels and higher-order modeling are discussed.

### 4.1. Virtual-Array DPD Example for Three Concurrent Users

Revisiting the example of Section 3.1, consider again the beam indices $\left\{b_{1},b_{2},b_{3}\right\}=\left\{1,2,5\right\}$, but now replace the physical array size L=16 with a virtual-array size of $L_{v}=13$. The processing steps then follow directly from the VA-DPD structure of Figure 2: precoding into the virtual antenna space, nonlinear DPD processing, and transformation into the virtual beam domain via an IFFT. This yields

(14) $$\begin{array}{c} L_{v}W_{\mathrm{DFT}}^{*}f\left(W_{\mathrm{DFT}}x\right)=\left[\begin{array}{c} \theta_{1}x_{1}+\frac{1}{L_{v}}\theta_{3}x_{1}\left(\right|x_{1}{|}^{2}+|x_{2}{|}^{2}+|x_{3}{|}^{2})\\ \theta_{1}x_{2}+\frac{1}{L_{v}}\theta_{3}x_{2}\left(\right|x_{1}{|}^{2}+|x_{2}{|}^{2}+|x_{3}{|}^{2})\\ \frac{1}{L_{v}}\theta_{3}x_{2}^{2}x_{1}^{*}\\ \frac{1}{L_{v}}\theta_{3}x_{1}x_{3}x_{2}^{*}\\ \theta_{1}x_{3}+\frac{1}{L_{v}}\theta_{3}x_{3}\left(\right|x_{1}{|}^{2}+|x_{2}{|}^{2}+|x_{3}{|}^{2})\\ \frac{1}{L_{v}}\theta_{3}x_{2}x_{3}x_{1}^{*}\\ 0\\ \frac{1}{L_{v}}\theta_{3}x_{3}^{2}x_{2}^{*}\\ \frac{1}{L_{v}}\theta_{3}x_{3}^{2}x_{1}^{*}\\ \frac{1}{L_{v}}\theta_{3}x_{1}^{2}x_{3}^{*}\\ \frac{1}{L_{v}}\theta_{3}x_{1}x_{2}x_{3}^{*}\\ \frac{1}{L_{v}}\theta_{3}x_{2}^{2}x_{3}^{*}\\ \frac{1}{L_{v}}\theta_{3}x_{1}^{2}x_{2}^{*} \end{array}\right]\;. \end{array}$$

instead of (12). Since $L_{v}<L$, fewer DPD computations have been performed whilst generating the same predistorted data streams as in (12). And since these data streams are separated, they can then easily be combined or precoded to an arbitrarily large physical array of size $L\geq L_{v}$ by merely adding zeros and rearranging the terms.

By combinatorial trials, i.e., computing (13) for all possible combinations of $\left\{b_{1},b_{2},b_{3}\right\}$, $L_{v}=13$ is found to be the smallest virtual domain that, for any *L*, produces all main and intermodulation streams separated as in (14) for K=3, P=3. Similarly, $L_{v}=4$ is the smallest such virtual domain for K=2 users, and $L_{v}=30$ for K=4. However, there are cases when the virtual domain can be made with an even smaller $L_{v}$, without loss of information. This will be explained further in the following subsection.

### 4.2. Special Beam Configuration Cases

Let $\left\{b_{1}^{\prime },b_{2}^{\prime },b_{3}^{\prime }\right\}$ denote the set of main beam indices in the virtual domain, with $\left\{b_{1},b_{2},b_{3}\right\}$ representing the corresponding user beam indices in the real array. Depending on the real beam configuration, $\left\{b_{1},b_{2},b_{3}\right\}$, and the physical array size, *L*, different choices of $\left\{b_{1}^{\prime },b_{2}^{\prime },b_{3}^{\prime }\right\}$ and $L_{v}$ may be used to obtain an optimally dimensioned virtual array. More exact details on the problem of finding a virtual subspace are found in Appendix A, and are detailed statistically in the results of Section 5.4. There, it is noted how all sets of $\left\{b_{1},b_{2},b_{3}\right\}$ can be represented with varying sets of $L_{v}$ and $\left\{b_{1}^{\prime },b_{2}^{\prime },b_{3}^{\prime }\right\}$.

**Figure 3:**
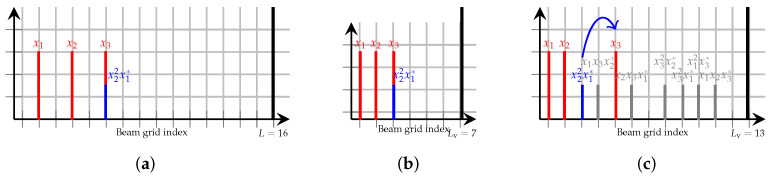
Beam occurrences for $\left\{b_{1},b_{2},b_{3}\right\}=\left\{2,4,6\right\}$ in real and virtual arrays, with placement of $x_{2}^{2}x_{1}^{*}$ highlighted. (**a**) Beam occurrences in real array. (**b**) Beam occurrences when $L_{v}=7$, $\left\{b_{1}^{\prime },b_{2}^{\prime },b_{3}^{\prime }\right\}=\left\{1,2,3\right\}$. (**c**) Beam occurrences when $L_{v}=13$, $\left\{b_{1}^{\prime },b_{2}^{\prime },b_{3}^{\prime }\right\}=\left\{1,2,5\right\}$.

The key phenomenon explaining why different sizes of $L_{v}$ can occur is array aliasing, as observed in Section 3.2, which can cause intermodulation terms to overlap. When this happens, the number of distinct IM directions is reduced, and consequently, the virtual domain required to represent them need not be as large. For example, evenly spaced beams in an even length array produce IM terms only at even beam indices. Concretely, say L=16 and $\left\{b_{1},b_{2},b_{3}\right\}=\left\{2,4,6\right\}$. Then, by (13), the beam indices for a third-order nonlinearity are [2,4,6,8,10,14,16], a total of seven unique beam directions. However, computing (13) with $L_{v}=7$, it can be noted that with $\left\{b_{1}^{\prime },b_{2}^{\prime },b_{3}^{\prime }\right\}=\left\{1,2,3\right\}$, the same IM terms will overlap with one another, but at indices [1,2,3,4,5,6,7]. This is illustrated in Figure 3a–c. In Figure 3a, placement of main beams on indices $\left\{b_{1},b_{2},b_{3}\right\}=\left\{2,4,6\right\}$ is shown, in the real array of size L=16. It is highlighted how this particular configuration results in the IM-term $x_{2}^{2}x_{1}^{*}$ overlapping with $x_{3}$ in the beam domain. Figure 3b then showcases how the same overlap of $x_{2}^{2}x_{1}^{*}$ and $x_{3}$ is obtained in the virtual beam domain, for a virtual array of size $L_{v}=7$. Should it hold that IM overlap is the same for all beams in the real array and the virtual array, then the virtual array can perfectly represent the nonlinear system.

In Figure 3c, it is shown that a larger virtual array (here $L_{v}=13$) can also be used to first generate all IM terms on distinct beam indices and then shift them to match the pattern of IM overlap in the real array.

In all, this subsection showcases that: (1) the virtual domain size can be made smaller in special cases and (2) the resulting virtual domain sizes in the proposed approach are, at worst, similar in size to the cardinality of unique main beam and IM beam directions.

### 4.3. Further Notes on Beamforming

For analytical clarity, this work has focused on LOS channels, wherein the columns of the DFT precoder matrix of (9) describe the inverse channel for transmission in those orthogonal LOS directions. However, more generally, if the received signal can be related to the wireless channel model as

(15) $$Y=Hf_{\mathrm{PA}}\left(z\right)\;,$$

then the method is still applicable with some modifications. Looking at the bar plots in Figure 2, and exemplified in (14), it is seen that the main and IM beam components are separated after the IFFT. Thus, the final block can be replaced with a different precoding matrix W. Presume for example that some general beamforming vector $w^{\left(1\right)}$ should be applied to spatially direct $x_{1}$, and $w^{\left(2\right)}$ to spatially direct $x_{2}$. Seen exemplified in (5), the third-order off-beam beamforming vectors will then be ${\left(w^{\left(1\right)}\right)}^{2}{\left(w^{\left(2\right)}\right)}^{*}$ and ${\left(w^{\left(2\right)}\right)}^{2}{\left(w^{\left(1\right)}\right)}^{*}$, for $x_{1}^{2}x_{2}^{*}$ and $x_{2}^{2}x_{1}^{*}$, respectively. Thus, the precoder in the final block could be computed with $W=\left[\begin{array}{cccc} w^{\left(1\right)} & w^{\left(2\right)} & {\left(w^{\left(1\right)}\right)}^{2}{\left(w^{\left(2\right)}\right)}^{*} & {\left(w^{\left(2\right)}\right)}^{2}{\left(w^{\left(1\right)}\right)}^{*} \end{array}\right]$ instead of through an FFT. A simple simulation case is shown in Section 5.6, with more complex cases left for future work.

Realistic channels would in practice also include phenomena such as fading, interference, and multi-path effects. This can require more sophisticated approaches to channel estimation and coding, and can be addressed in many different ways, e.g., as found in [19,20]. Practical precoding strategies, such as the Enhanced Type II codebook in 5G NR in 3GPP, will in many complex channel scenarios often rely on a grid of beams, but in addition consider frequency domain information in the processing [21,22]. This is based on the observation that any realistic channel can be decomposed into the sum of orthogonal beams. Additionally, such codebooks feature oversampling of the DFT beamgrid, allowing flexible beam selection for users who would otherwise fall in between beams on a regular beamgrid. Depending on the practical scenario, the spatial sparsity of the channel can take one of three forms for the precoder. Consider this for a tri-user scenario. Let *B* denote the unique number of beam directions present due to nonlinearity. In the first form, the precoding scheme decides that the three users can be perfectly served with three main beams, and B−3 off-beams, on the DFT beamgrid. This form is optimally sparse and can yield the largest possible reduction in complexity for VA-DPD, since IM beams may overlap and thereby reduce *B*. In the second form, the precoder decides that three main beams are dominant in serving the users, although performance can be improved by injecting weak signals on other beam indices as well. In this case, the VA-DPD can decide to only consider the dominant beams for linearization, whilst IM from low-magnitude beams is neglected. This gives form one, but by approximation. In the third form, the precoder decides that power needs to be distributed evenly across several beams for each respective user. In this case, the VA-DPD can treat the sum of these beamgrid vectors as one effective precoding vector. For example, if the first user needs signal injection on beam indices $\left\{b_{1},b_{3},b_{7}\right\}$ with respective magnitudes of 0.27,0.33,0.4, then the joint precoding vector can analytically be considered as $\omega^{\left(1\right)}=0.27\omega^{\left(b_{1}\right)}+0.33\omega^{\left(b_{3}\right)}+0.4\omega^{\left(b_{7}\right)}$. As such, the earlier analysis of this subsection can be used.

Finally, advances in MIMO beamforming research include concepts such as cooperative networks [23], as well as content-centric beamforming rather than user-centric beamforming [24]. For the latter, the VA-DPD framework can be applied without any loss of generality by interpreting $x_{1},\ldots ,x_{K}$ as *K* content streams rather than user streams.

### 4.4. Generalization to Higher-Order Terms

The analysis so far has focused on third-order IM terms, but extension to higher-order nonlinearities is straightforward. By augmenting the PA model in (4) with higher-order terms and repeating the derivations, additional IM products appear. For instance, including a fifth-order term in the PA model gives rise to IM components such as $x_{3}^{3}x_{1}^{*}x_{2}^{*}$, which, following the same indexing rules as before, appear at beam index $\bar{3b_{3}-b_{1}-b_{2}}$. As for the third-order IM terms, some of these higher-order IM terms will always be directed towards the users, and some will be off-beams. Considering all possible high-order IM beams, the beam domain is only sparse for larger *L*, and for VA-DPD to guarantee complete equivalence to PP-DPD for any beam configuration, $L_{v}$ must be increased accordingly. However, the fifth-order IM terms will typically have significantly lower power than the first- and third-order terms. Thus, they can often be neglected in off-beam directions. At the end, it is also a trade-off between DPD complexity costs against PA efficiency gains that dictates how nonlinear the PAs should be driven.

### 4.5. Analogy with Frequency Relocation

**Figure 4:**
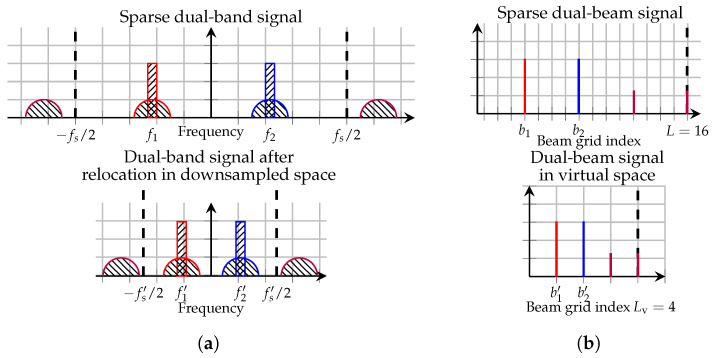
Comparison of a dual-band frequency relocation approach for densifying the frequency spectrum to the virtual-array approach for densifying the beam domain. (**a**) Power spectral density of a dual-band signal passed through a nonlinearity, with induced IM, in the frequency domain. (**b**) Power of a multi-beam signal passed through a nonlinearity, with induced IM, in the beam domain.

Whilst not a direct counterpart, the proposed VA-DPD approach bears conceptual similarities to frequency relocation as in [11]. Namely, a signal that is sparse in one of its domains is densified in that domain prior to DPD processing. In the upper part of Figure 4a, a sparse dual-band signal, with the respective bands centered on frequencies $f_{1}$ and $f_{2}$ and sampled with rate $f_{s}$, is shown. IM terms here appear outside the first Nyquist zone. It is then, in the lower part, illustrated how the frequency domain can be densified by relocating the bands to frequencies $f_{1}^{\prime }$ and $f_{2}^{\prime }$, thus enabling a sample rate reduction to $f_{s}^{\prime }$. The DPD processing is then carried out at this lower rate before the frequency shift is reversed.

Figure 4b illustrates the corresponding spatial interpretation. The upper part shows the DFT beam-domain representation of an *L*-element array transmitting two beams at indices $b_{1}$ and $b_{2}$, along with their associated IM components. In the lower part, these beams (and their IM products) are mapped onto a smaller virtual beamgrid of size $L_{v}$, creating a densified representation of the same spatial content. Instead of allowing a sample rate reduction, the corresponding gain is that it allows fewer DPDs to linearize a larger array.

A challenge in multi-band frequency relocation is when bands are unevenly spaced, giving rise to IM products that end up at new frequency locations. In such cases, in [11], uniform downsampling whilst maintaining the relative spacing of bands is performed. This, however, will often not densify the frequency spectrum to only contain the relevant bands and IM products. In contrast, VA-DPD can in the multi-beam setting achieve a greater degree of dimensionality reduction by careful choice of the relocated beam indices in the virtual domain.

## 5. Numerical Setup and Results

For the numerical simulations, MATLAB (v2025b) is used. A setup with L=28 or 64 antennas, depending on the subsection, in a uniform linear array serving K=2 or 3 users, is considered. Each PA is modeled with a baseband Saleh model [25], equal on each PA branch, with added Gaussian noise $n_{l}$ at the output as

(16) $$f_{\mathrm{PA},l}\left(z_{l}\right)=\frac{az_{l}}{1+b|z_{l}{|}^{2}}+n_{l}\;,$$

with a=1,b=0.47. The desired signal is set to

(17) $$Y_{d}=a_{d}Hz\;,$$

with $a_{d}=0.95$. The noise power of $n_{l}$ is chosen to give signal-to-noise ratios between 50 and 60 dB. H is chosen as an LOS channel, from *L* branches to Q=181 evenly spaced observation directions in the azimuth plane. Each channel element is $h_{i_{1},i_{2}}=\exp\left(j2\pi i_{1}d\cos\left(\phi_{i_{2}}\right)/\lambda \right)$, where *d* denotes the array antenna spacing and λ the wavelength, with d/λ=0.5, $\phi_{i_{2}}$ is the azimuth angle, and $i_{1}\in \left[-L/2,-L/2+1,\ldots ,L/2-1\right]+1/2,\;i_{2}\in \left[1,Q\right]$. The baseband user data streams are generated through bandlimiting complex Gaussian noise to a bandwidth of 30 MHz, and normalized to a root mean square value of 0.13, with a peak-to-average power ratio of about 8.3 dB. For each user stream, *N* = 655,36 baseband samples are generated.

Four linearization cases are simulated per transmit configuration: not using DPD (No DPD), using per-PA (PP) DPD, using beam-domain (BD) DPD based on [5,6], and using virtual-array (VA) DPD.

### 5.1. DPD Identification

With this setup, conventional iterative learning control (ILC) [26] is modified with a simple extension and used to find the optimal input signals $z_{l}$ to each PA in the array. Let

(18) $$z^{\left[i\right]}=\left[\begin{array}{c} z_{1}^{\left[i\right]}\\ \vdots \\ z_{L}^{\left[i\right]} \end{array}\right]\;.$$

denote the DPD input at iteration [i]. Then, the DPD input at iteration [i+1], $z^{\left[i+1\right]}$, based on the OTA measured $Y^{\left[i\right]}$ and desired signal $Y_{d}$, is found as

(19) $$z^{\left[i+1\right]}\leftarrow z^{\left[i\right]}+\eta H^{†}\left(Y_{d}-Y^{\left[i\right]}\right)\;,$$

Here, η is a step parameter for the iterations, $H^{†}={\left(H^{*}H\right)}^{-1}H^{*}$ is the pseudoinverse of the channel matrix H, and * denotes the Hermitian transpose. This ILC procedure is suitable for performing offline as an initial identification, whilst online adaptation of DPD coefficients can be done with other known methods. The signals $z_{l}$ are, after ILC convergence, shifted to the virtual domain, yielding target signals $z_{l}^{\prime }$, as in Figure 2. The shifting is performed by doing the VA processing steps in reverse. That is, an IFFT is performed on z along the branches, followed by relocating the streams of different considered beams from z to their VA placement $\left\{b_{1}^{\prime },\ldots ,b_{B}^{\prime }\right\}$, whilst removing streams that were zero-padded in the forward path. Lastly, an FFT is done in the virtual domain to go from the virtual beam domain to the virtual antenna domain and obtain $z_{l}^{\prime }$. Forming a nonlinear model from $u_{l}^{\prime }$ to $z_{l}^{\prime }$ in the virtual domain through polynomial DPD models yields a predistorted virtual domain signal and basis

(20) $$z_{l}^{\prime }=F_{u_{l}^{\prime }}\theta_{l}\;,F_{u_{l}^{\prime }}=\left[\begin{array}{ccc} u_{l}^{\prime }, & u_{l}^{\prime }{\left|u_{l}^{\prime }\right|}^{2}, & \ldots u_{l}^{\prime }{\left|u_{l}^{\prime }\right|}^{P-1} \end{array}\right]$$

where DPD coefficients are obtained as

(21) $$\theta_{l}=F_{u_{l}^{\prime }}^{†}z_{l}^{\prime }\;.$$

For conventional PP-DPD, the same relations (20) and (21) are used, but with target signals $z_{l}$ and inputs $u_{l}$ in the original antenna domain (as in Figure 1). For BD-DPD, each unique basis function expressed in terms of $x_{1},\ldots ,x_{K}$, obtainable from the expansion of $\sum_{p=0,2,\ldots }^{P-1}z_{l}{\left|z_{l}\right|}^{p}$, is used. This includes off-beam terms as in Table 1, fifth-order terms as in Table 2, and main beam terms such as $x_{1}|x_{1}{|}^{2},x_{2}{\left|x_{3}\right|}^{2}$.

### 5.2. Complexity Analysis

To quantitatively compare BD-DPD and VA-DPD, this subsection provides a brief complexity analysis. It limits its scope to the filtering complexity, as the initial identification can be done offline, and as the filtering complexity typically dominates over the adaptation complexity [5,27].

Consider first the VA-DPD scheme in Figure 2. The computational complexity of the scheme consists of four parts: the VA FFT precoding, VA DPDs, the IFFT, and precoding back to the real array. The first step maps *K* user streams to $L_{v}$ virtual branches using an $L_{v}$-point FFT. The complexity $F_{\mathrm{FFT}}\left(L_{v}\right)$ depends on the FFT size and implementation:

- $L_{v}=4$: A radix-4 FFT requires 16 real additions $\Rightarrow F_{\mathrm{FFT}}\left(4\right)=16$ FLOPs.
- $L_{v}=13$ (prime): A Winograd 13-point FFT requires 42 real multiplications and 188 real additions, $\Rightarrow F_{\mathrm{FFT}}\left(13\right)=230$ FLOPs.
- $L_{v}=30$: A Good–Thomas FFT exploiting 2×3×5=30 using a two-point FFT + Winograd-3 and Winograd-5 kernels: 50 additions + 22 multiplications, $\Rightarrow F_{\mathrm{FFT}}\left(30\right)=\;576$ FLOPs [28].

These costs are reported separately from the DPD complexity, as FFT implementation in hardware may differ significantly from DPD processing. In cases where $F_{\mathrm{FFT}}\left(L_{v}\right)$ is high, it may even be beneficial to increase $L_{v}$ to reduce the FFT cost at the expense of a slight increase in DPD complexity. The final IFFT has the same cost and is therefore included as a factor of 2 in Table 3.

As for the VA-DPD units, complexity will scale corresponding to the number of basis functions and virtual branches as $B_{\mathrm{basis}}L_{v}$. For an odd-order memory polynomial (MP) of nonlinearity order *P* and memory *M*, $B_{\mathrm{basis}}=4\left(P+1\right)\left(M+1\right)$ [27]. Finally, the precoder goes from $L_{v}$ virtual branches to *L* real branches. However, this complexity is omitted from comparison, as precoding to the real array must be performed for all DPD methods, to approximately the same computational cost.

The filtering complexity for BD-DPD implementing an MP model is $8\left(M+1\right)\sum_{p=1,3,\ldots }^{P}\left|\kappa_{p}\right|$, where $\kappa_{p}$ denotes the cardinality of the set $\kappa_{p}$, i.e., the number of bases for nonlinearity terms of order *p*. Only odd-order polynomials are considered. For example, for a two-user case, $\kappa_{5}=12$, as seen from Table 2. After the multi-dimensional DPD, a precoder of dimension *L* follows, as in the VA-DPD case.

The filtering complexity for an MP PP-DPD follows as 4(P+1)(M+1)L. Table 3 summarizes the comparison. In Table 3, the number of coefficients necessary for simple lookup table implementations is provided as well, based on [29]. That is, whilst each VA-DPD and PP-DPD branch can adapt a 1D LUT per branch, as

(22) $$z_{l}\left[n\right]=\sum_{m=0}^{M-1}u_{l}\left[n-m\right]f_{\mathrm{LUT}}\left(\right|u_{l}\left[n-m\right]\left|\right)\;,$$

beam-domain approaches in direct extension need to consider multi-dimensional LUTs. For example, for the first main beam, they are of the form

(23) $$u_{1}\left[n-m\right]=\sum_{m=0}^{M-1}x_{1}\left[n-m\right]f_{\mathrm{LUT}}\left(\right|x_{1}\left[n-m\right]|,\ldots ,|x_{K}\left[n-m\right]\left|\right)\;.$$

Correspondingly, LUTs for off-beam IM terms also need to be adapted, exemplified as

(24) $$\begin{array}{cc} u_{i}\left[n-m\right]=\sum_{m=0}^{M-1}\; & x_{1}\left[n-m\right]x_{2}\left[n-m\right]x_{3}^{*}\left[n-m\right]\cdot \\ \; & f_{\mathrm{LUT}}\left(\right|x_{1}\left[n-m\right]|,|x_{2}\left[n-m\right]|,|x_{3}\left[n-m\right]\left|\right)\;, \end{array}$$

where i∈[1,B]. For PP-DPD and VA-DPD, each branch requires (M+1) one-dimensional LUTs. Denote the LUT depth as *T*. Then, VA-DPD requires $\left(M+1\right)TL_{v}$ coefficients, whilst PP-DPD requires (M+1)TL. For BD-DPD, a multi-dimensional LUT per unique beam is necessary prior to the precoder. Compared to Table 1, it is noted that off-beams will not always need *K*-dimensional LUTs. Instead, the necessary number of LUT coefficients can be computed as $K\left(M+1\right)T^{K}+\sum_{i=0}^{K-2}B_{i}{\left(M+1\right)}^{K-i}$. Here, $B_{i}$ is the number of off-beam terms requiring (K−i)-dimensional LUTs.

As a final note, it is worth mentioning that the introduction of FFT and IFFT blocks in the VA-DPD is expected to introduce only minor latency, as the FFT lengths are small.

### 5.3. Tri-Beam Scenario

**Figure 5:**
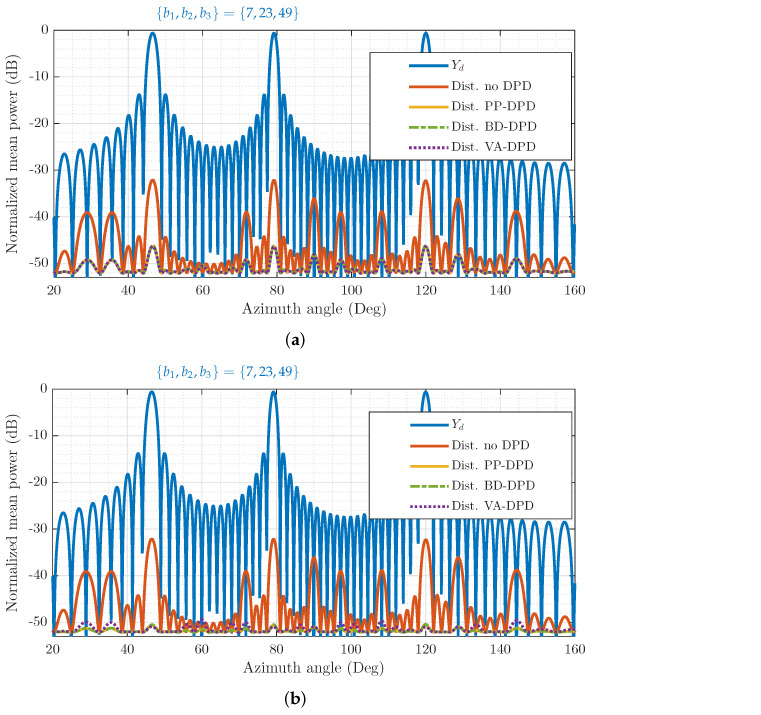

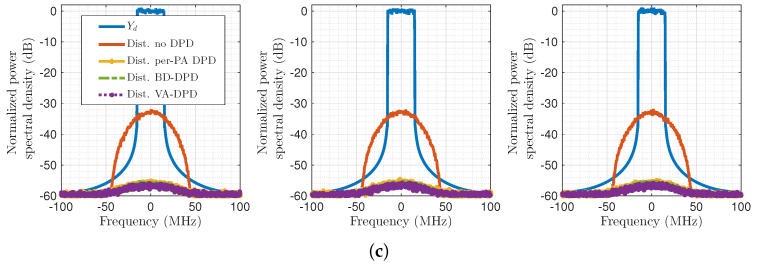
Normalized power of the desired signal $Y_{d}$ shown in comparison to the distortion power remaining after no DPD, per-PA (PP) DPD, the proposed virtual-array (VA) DPD, and beam-domain (BD) DPD, for concurrent tri-beam transmission. DPDs include odd-order terms up to, and including, order *P*. (**a**) Mean power in varying spatial directions, P=3. (**b**) Mean power in varying spatial directions, P=5. (**c**) Power spectral density in user directions, P=5.

In Figure 5, a concurrent tri-beam scenario is shown. For this scenario, there are L=64 transmit branches, with beams in beam directions $\left\{b_{1},b_{2},b_{3}\right\}=\left\{7,23,49\right\}$. In Figure 5a,b, the power of the desired signal $Y_{d}$, over different angles, is visualized in contrast to the distortion power remaining after applying no DPD, PP-DPD, BD-DPD, and the proposed VA-DPD approach with either odd-order P=3 or P=5 DPDs. For VA-DPD, the virtual array uses $L_{v}=13$ branches, meaning it can capture third-order beam direction information without loss. Consequently, Figure 5a highlights how VA-DPD is equivalent in performance to PP-DPD and BD-DPD for a DPD of order P=3. This is seen from the fact that the yellow line, corresponding to PP-DPD distortion, is precisely covered by the purple and green ones, of VA-DPD and BD-DPD, respectively. On the other hand, compared to Figure 5a, Figure 5b shows that applying fifth-order DPDs whilst only considering up to third-order terms in the virtual-array dimensioning reduces the distortion power in the user directions equivalently to PP-DPD and BD-DPD, by an additional 5 dB compared to P=3. However, the off-beam linearization performance is not improved correspondingly, as seen by the residual distortion, e.g., around angles 28–35°. The power spectral density of the desired signal in the user directions and the residual error after linearization are shown for the respective methods in Figure 5c. The normalized mean square error (NMSE) between the desired and received user signal is about −50 dB for each method in all user directions, for P=5.

With $L_{v}=13$ branches, no memory, and P=5, Table 3 gives a DPD unit complexity of 312 FLOPs, and $2F_{\mathrm{FFT}}\left(13\right)=460$ FLOPs. Notably, for such simple DPD units, the FFT complexity is large. However, for more complex DPDs, the impact is relatively diminished. Alternatively, for the DPD units, $L_{v}=13$ 1D-LUT lookups are done to find the appropriate predistorted values per sample. Assuming a LUT depth of T=10, this gives 130 LUT entries. In contrast, PP-DPD requires 1536 FLOPs or 640 LUT entries. Thus, the DPD unit complexity is reduced by 100·|312−1536|/1536≈80% in VA-DPD compared to PP-DPD, though the FFTs also need to be accounted for. Lastly, as seen from Table 1 and Table 2, BD-DPD needs to account for 3 linear, 12 third-order and 60 fifth-order terms. Table 3 shows that with B=12 unique data streams, BD-DPD requires 648 FLOPs or 6600 LUT entries.

### 5.4. Virtual-Array Dimensioning

**Figure 6:**
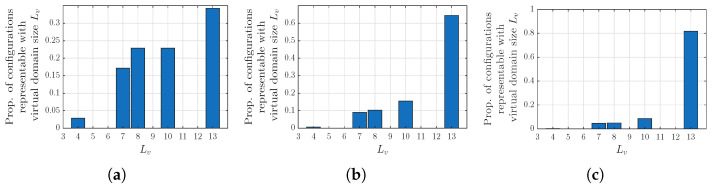
Proportion of main beam configuration cases in the original domain of size *L* that can be represented by a virtual domain of size $L_{v}$, considering IM terms of order P=3. (**a**) Array size L=16. (**b**) Array size L=32. (**c**) Array size L=64.

The results of this section describe how small the virtual array can be dimensioned in terms of $L_{v}$, to account for all IM beams, with respect to special cases as described in Section 4.2. In Figure 6, the proportion of beam configuration $\left\{b_{1},b_{2},b_{3}\right\}$ that can be represented with a virtual array of length $L_{v}$ is shown for varying array sizes L=16,32,64, and in Table 4 and Table 5, it is shown for various *L* and P=3,5. Most noticeably, the proportion of cases requiring a larger $L_{v}$ increases with *L*. This correlates to the fact that in a larger beam space, the IM products end up in unique beam directions more often. The results highlight that there are cases when the beam domain is especially sparse, such that the VA-DPD can be performed at an even lower computational complexity.

### 5.5. Power Amplifier Variations

**Figure 7:**
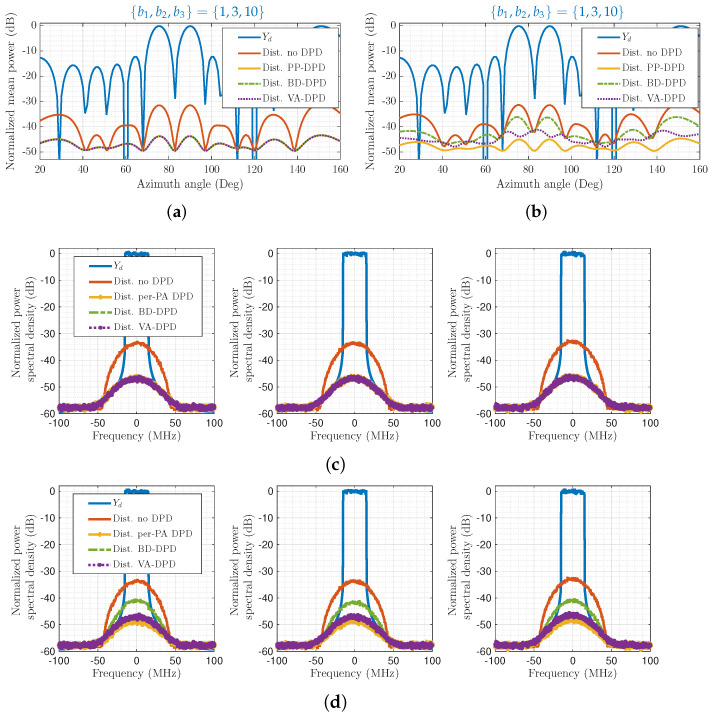
Normalized power of the desired signal $Y_{d}$ shown in comparison to the distortion power remaining after no DPD, per-PA (PP) DPD, the proposed virtual-array (VA) DPD, and beam-domain (BD) DPD, for concurrent tri-beam transmission, with or without PA variations. DPDs include first- and third-order terms. (**a**) Mean power in varying spatial directions. No PA variations. (**b**) Mean power in varying spatial directions. PA variations. (**c**) Power spectral density in user directions. No PA variations. (**d**) Power spectral density in user directions. PA variations.

It was noted earlier that BD-DPD assumes equal PAs across branches. It is this assumption that mathematically allows for a common DPD to be used prior to the precoder in BD-DPD, without losing any ability to linearize the system [6]. This assumption is not quite as rigidly held in the proposed VA-DPD, as DPD variations across the virtual-array branches can be adapted. As such, to investigate whether or not VA-DPD might handle PA variations better in comparison to BD-DPD, the PA model is changed to

(25) $$f_{\mathrm{PA},l}\left(x\right)=c_{1,l}x+c_{3,l}x{\left|x\right|}^{2}\;,$$

to more easily represent gain and phase variations in the nonlinear PA response relative to the linear response, variations which cannot be compensated by calibration. The first-order coefficients are set to $c_{1,l}=1$ for all branches, whilst $c_{3,l}$ are varied from a nominal value of −0.55 with magnitude standard deviation 0.1 and random phases from a uniform distribution in the range of ${\left[-20,20\right]}^{\circ }$. Figure 7 showcases a tri-beam case with $\left\{b_{1},b_{2},b_{3}\right\}=\left\{1,3,10\right\}$, L=16, $L_{v}=7$. In Figure 7a,c, the PAs exhibit no variations, whilst in Figure 7b,d, they do. Notably, both VA-DPD and BD-DPD deteriorate in performance relative to PP-DPD with PA variations. However, VA-DPD reduces distortion more than BD-DPD in all user directions. This is because, whilst BD-DPD adapts one set of coefficients prior to the precoder to hold for linearization across all branches, VA-DPD can have some DPD coefficient variations with $l=1,\ldots ,L_{v}$. For VA-DPD, the NMSE is about −42 dB in each user direction when no PA variations are present, and about −40 dB with PA variations. The corresponding values for PP-DPD are −42 dB and −43 dB, and for BD-DPD are −42 dB and −36 dB.

For both BD-DPD and VA-DPD, additional consideration of PA variations across branches can be necessary, depending on the severity of the PA variations. One approach of mitigation is to account for variations through minor DPD computations at each branch, whilst the main part of predistortion is still performed as per the respective method, similar to [4].

### 5.6. Non-FFT Precoder

**Figure 8:**
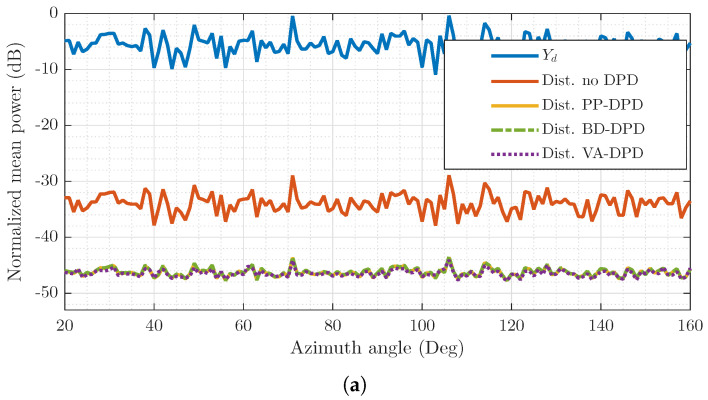

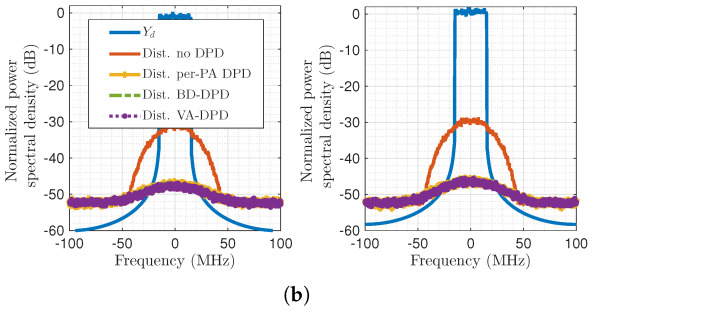
Normalized power of the desired signal $Y_{d}$ shown in comparison to the distortion power remaining after no DPD, per-PA (PP) DPD, the proposed virtual-array (VA) DPD, and beam-domain (BD) DPD, for concurrent dual-beam transmission in a random channel. P=7 is used for the DPDs. (**a**) Mean power in varying spatial directions. (**b**) Power spectral density in user directions.

In Figure 8, a two-user scenario for a random channel H of dimension L×Q consisting of complex Gaussian numbers distributed as CN(0,1) is shown, with users in directions ${\left[71,106\right]}^{\circ }$. The array is of size L=28, and virtual array of size $L_{v}=4$. Precoding vectors are chosen as the pseudoinverse of the respective channel vectors, i.e., $w_{1}=H_{71}^{†},$$\;w_{2}=H_{106}^{†}$. First, it is important to note that all linearization methods achieve about 20 dB improvement in distortion reduction. Secondly, neither the linear power nor the residual distortion forms clear beam directions but is instead spread across the spatial range due to the randomness of the channel. From Table 3, VA-DPD has a DPD unit filtering complexity of 128 FLOPs for P=7,M=0, $2F_{\mathrm{FFT}}\left(4\right)=32$ FLOPs, and requires $L_{v}T=4\cdot 10=40$ coefficients for LUTs of size 10. Correspondingly, BD-DPD requires 256 FLOPs and 220 LUT coefficients, whilst PP-DPD costs 896 FLOPs and would require 280 LUT coefficients. Thus, a 100·|156−896|/896≈83% reduction in DPD unit complexity is achieved for VA-DPD compared to PP-DPD. For all DPD methods, an NMSE of about −40 dB is obtained in each user direction.

## 6. Conclusions

The proposed DPD schematic, using FFT beamforming and virtual-array DPD processing, is shown to be capable of reducing DPD complexity manyfold in contrast to conventional per-PA DPD, when many transmit branches serve few users. By theoretical derivation and simulation, it is shown how an arbitrarily large array can be linearized by no more than 4, 13, or 30 virtual-array branches when only 2, 3, or 4 users, respectively, are served. The virtual-array DPD scheme is shown to be operable for varying degrees of PA nonlinearities, and for different channel conditions and multi-user scenarios. In contrast to previous approaches utilizing a beam-domain framework, the proposed approach utilizes single-input, single-output DPD units that are computationally cheap to implement. Additionally, it is shown how VA-DPD can adapt to PA variations better than beam-domain-oriented solutions. Overall, the approach offers a flexible and powerful alternative to multi-user linearization for large MIMO arrays with digital beamforming.

Future work will include experimental validation and studying practical concerns such as FPGA implementation feasibility and other real-time constraints. Additionally, the power consumption trade-off between PA back-off and DPD complexity warrants further study.

## Figures and Tables

**Table 1:** Off-beam terms for static nonlinearity of order P=3, and with K=2,3,4 concurrent users.

Number of Users	Off-Beam Terms	Number of Off-Beam Terms
K=2	$x_{1}x_{1}x_{2}^{*}$, $x_{2}x_{2}x_{1}^{*}$	2
K=3	$x_{1}x_{1}x_{2}^{*}$, $x_{2}x_{2}x_{1}^{*}$, $x_{1}x_{1}x_{3}^{*}$, $x_{3}x_{3}x_{1}^{*}$ $x_{2}x_{2}x_{3}^{*}$, $x_{3}x_{3}x_{2}^{*}$, $x_{1}x_{2}x_{3}^{*}$, $x_{1}x_{3}x_{2}^{*}$, $x_{2}x_{3}x_{1}^{*}$	9
K=4	$x_{1}x_{1}x_{2}^{*}$, $x_{2}x_{2}x_{1}^{*}$, $x_{1}x_{1}x_{3}^{*}$, $x_{3}x_{3}x_{1}^{*}$ $x_{2}x_{2}x_{3}^{*}$, $x_{3}x_{3}x_{2}^{*}$, $x_{1}x_{2}x_{3}^{*}$, $x_{1}x_{3}x_{2}^{*}$, $x_{2}x_{3}x_{1}^{*}$, $x_{1}^{2}x_{4}^{*}$, $x_{4}^{2}x_{1}^{*}$, $x_{2}^{2}x_{4}^{*}$, $x_{4}^{2}x_{2}^{*}$, $x_{3}^{2}x_{4}^{*}$, $x_{4}^{2}x_{3}^{*}$, $x_{1}x_{2}x_{4}^{*}$, $x_{1}x_{4}x_{2}^{*}$, $x_{2}x_{4}x_{1}^{*}$, $x_{1}x_{3}x_{4}^{*}$, $x_{1}x_{4}x_{3}^{*}$, $x_{3}x_{4}x_{1}^{*}$, $x_{2}x_{3}x_{4}^{*}$, $x_{2}x_{4}x_{3}^{*}$, $x_{3}x_{4}x_{2}^{*}$	24

**Table 2:** Fifth-order IM terms for static nonlinearity of order P=5, with K=2,3 concurrent users.

Number of Users	Fifth-Order Terms	Total Number of Fifth-Order Terms
K=2	$x_{1}{\left|x_{1}\right|}^{4}$, $x_{1}{\left|x_{2}\right|}^{4}$, $x_{1}|x_{1}{|}^{2}{\left|x_{2}\right|}^{2}$ $x_{2}{\left|x_{2}\right|}^{4}$, $x_{2}{\left|x_{1}\right|}^{4}$, $x_{2}|x_{1}{|}^{2}{\left|x_{2}\right|}^{2}$ $x_{1}^{3}{\left(x_{2}^{*}\right)}^{2}$, $x_{2}^{3}{\left(x_{1}^{*}\right)}^{2}$ $x_{1}{\left|x_{1}\right|}^{2}x_{2}^{2}$, $x_{2}{\left|x_{2}\right|}^{2}x_{1}^{2}$ $x_{1}^{2}{\left|x_{1}\right|}^{2}x_{2}^{*}$, $x_{2}^{2}{\left|x_{2}\right|}^{2}x_{1}^{*}$	12
K=3	$x_{1}{\left|x_{1}\right|}^{4}$, …, $x_{1}|x_{2}{|}^{2}{\left|x_{3}\right|}^{2}$	60

**Table 3:** DPD filtering complexity for the different schemes, assuming implementation of memory-polynomial bases, given in the number of floating-point operations (FLOPs) per baseband sample. The number of lookup (LUT) table entries is also listed, if DPDs are implemented as LUTs instead. *T* is the LUT size. The virtual-array FFT/IFFT complexity is listed as $F_{\mathrm{FFT}}\left(L_{v}\right)$.

Operation	Per-PA DPD (FLOPs)/(# LUT Entries)	Beam-Domain DPD (FLOPs)/(# LUT Entries)	Virtual-Array DPD (FLOPs)/(# LUT Entries)
FFT/IFFT	NA/NA	NA/NA	$2F_{\mathrm{FFT}}\left(L_{v}\right)$/NA
DPD computation	4(P+1)(M+1)L/ (M+1)TL	$8\left(M+1\right)\sum_{p=1,3,\ldots }^{P}\left|\kappa_{p}\right|$/ $K\left(M+1\right)T^{K}+\sum_{i=0}^{K-2}B_{i}{\left(M+1\right)}^{K-i}$	$4\left(P+1\right)\left(M+1\right)L_{v}$/ $\left(M+1\right)TL_{v}$

**Table 4:** Percentage (%) of main beam configurations in the original domain of size *L* that can be represented by a virtual domain of size $L_{v}$, for IM terms of order P=3.

	$L_{v}$	3	4	5	6	7	8	9	10	12	13
*L*											
20		0	2	4	0	11	14	0	21	0	49
24		<1	1	0	4	10	12	0	14	21	38
30		<1	0	2	2	7	9	0	15	18	47
36		<1	1	0	2	7	7	3	11	12	58
130		0	0	≪1	0	2	2	0	5	0	91
256		0	≪1	0	0	1	1	0	2	0	95

**Table 5:** Percentage (%) of main beam configurations in the original domain of size *L* that can be represented by a virtual domain of size $L_{v}$, for IM terms of order P=5.

	$L_{v}$	4	8	11	12	16	21	22	24	26	30
*L*											
32		1	4	8	8	23	10	5	21	10	10
64		<1	1	4	4	10	7	4	15	7	47
128		≪1	<1	2	2	5	4	2	8	4	71

## Data Availability

The data generated in this work, described in Section 5, can be reproduced with the MATLAB function randn.

## Appendix A. Finding a Virtual DPD Space

Finding a virtual-array representation for a given array of size *L* transmitting beams $b_{1},\ldots ,b_{K}$ involves finding an appropriate virtual-array size $L_{v}$ and virtual beam placement $b_{1}^{\prime },\ldots ,b_{K}^{\prime }$. In the original antenna domain, for a tri-beam case, main and intermodulation beams will end up on integer indices $c_{1},\ldots ,c_{12}$, as in

(A1) $$\begin{array}{c} b_{1}=c_{1}\\ b_{2}=c_{2}\\ b_{3}=c_{3}\\ (2b_{1}-b_{2})-L\lfloor \frac{2b_{1}-b_{2}-1/2}{L}\rfloor =c_{4}\\ \vdots \\ (b_{2}+b_{3}-b_{1})-L\lfloor \frac{b_{2}+b_{3}-b_{1}-1/2}{L}\rfloor =c_{12}\;, \end{array}$$

where $\left\{c_{1},\ldots ,c_{12}\right\}\in \left[1,L\right]$. The set $\left\{c_{1},\ldots ,c_{12}\right\}$ might not be a unique set of 12 integers. This can be exemplified with beams $\left\{b_{1},b_{2},b_{3}\right\}=\left\{1,2,4\right\},\;L=16$, where the off-beam indices are {16,3,14,7,16,6,15,3,5} such that (A1) becomes

(A2) $$\begin{array}{cc} b_{1} & =c_{1}=1\\ b_{2} & =c_{2}=2\\ b_{3} & =c_{3}=4\\ (2b_{1}-b_{2})- & L\lfloor \frac{2b_{1}-b_{2}-1/2}{L}\rfloor =c_{4}=c_{8}=16\\ (2b_{2}-b_{1})- & L\lfloor \frac{2b_{2}-b_{1}-1/2}{L}\rfloor =c_{5}=c_{11}=3\\ \; & \vdots \\ (b_{1}+b_{3}-b_{2})- & L\lfloor \frac{b_{1}+b_{3}-b_{2}-1/2}{L}\rfloor =c_{11}=c_{5}=3\\ (b_{2}+b_{3}-b_{1})- & L\lfloor \frac{b_{2}+b_{3}-b_{1}-1/2}{L}\rfloor =c_{12}=5\;, \end{array}$$

The objective is then to find a virtual subspace with $L_{v},b_{1}^{\prime },b_{2}^{\prime },b_{3}^{\prime }$ such that

(A3) $$\begin{array}{cc} b_{1}^{\prime } & =c_{1}^{\prime }\\ b_{2}^{\prime } & =c_{2}^{\prime }\\ b_{3}^{\prime } & =c_{3}^{\prime }\\ (2b_{1}^{\prime }-b_{2}^{\prime })- & L_{v}\lfloor \frac{2b_{1}^{\prime }-b_{2}^{\prime }}{L_{v}}\rfloor =c_{4}^{\prime }=c_{8}^{\prime }\\ (2b_{2}^{\prime }-b_{1}^{\prime })- & L_{v}\lfloor \frac{2b_{2}^{\prime }-b_{1}^{\prime }}{L_{v}}\rfloor =c_{5}^{\prime }=c_{11}^{\prime }\\ \; & \vdots \\ (b_{1}^{\prime }+b_{3}^{\prime }-b_{2}^{\prime })- & L_{v}\lfloor \frac{b_{1}^{\prime }+b_{3}^{\prime }-b_{2}^{\prime }}{L_{v}}\rfloor =c_{11}^{\prime }=c_{5}^{\prime }\\ (b_{2}^{\prime }+b_{3}^{\prime }-b_{1}^{\prime })- & L_{v}\lfloor \frac{b_{2}^{\prime }+b_{3}^{\prime }-b_{1}^{\prime }}{L_{v}}\rfloor =c_{12}^{\prime }\;, \end{array}$$

for any $\left\{c_{1}^{\prime },\ldots ,c_{12}^{\prime }\right\}\in \left[1,L_{v}\right]$. This is a Diophantine system of equations and is generally not trivial to solve. However, in practice, it can be solved offline, and the indices can be obtained during runtime using a lookup table. In this work, the pseudo-algorithm of Algorithm A1 describes one approach to solving the systems of equations, for a general case with an arbitrary number of beams *K* and branches *L*. Depending on *K*, heuristic knowledge can limit the search space Lvs and mbs in Algorithm A1. One general constraint is $L_{v}\geq \left|\left\{b_{1}^{\prime },b_{2}^{\prime },\ldots ,b_{2}^{\prime }+b_{3}^{\prime }-b_{1}^{\prime }\right\}\right|$; i.e., the virtual domain size can never be reduced below the cardinality of the set it is supposed to represent.

**Algorithm A1** Algorithm to find virtual space representation.
**Require:** mb, *L* // Main beam indices mb and array size L // Get off-beams, and cardinality of full index set ob = get_offbeams(mb,L) ab = [mb ob] c = length(unique(ab)) // Try virtual subspace size Lv out of candidates Lvs **for** Lv=Lvs **do** // Try virtual main beams mbv out of candidates mbvs **for** mbv=mbvs **do** // Get off-beam indices for mbv obv = get_offbeams(mbv,Lv) abv = [mbv obv] // Relabel ab and abv such that the first element, and all other elements in the vector with the same value, is labelled 1. Do correspondingly for the remaining elements. Obtain ab_relabel, abv_relabel **if** ab_relabel==abv_relabel **then** // Virtual array representation {mbv,Lv} found. **end if** **end for** **end for**

## References

[1] Kakkavas A., Seco-Granados G., Wymeersch H., Garcia M.H.C., Stirling-Gallacher R.A., Nossek J.A. (2019). 5G Downlink Multi-Beam Signal Design for LOS Positioning. Proceedings of the IEEE Global Communications Conference.

[2] Liu X., Chen W., Chu J., Ghannouchi F.M., Feng Z. (2021). Multi-Stream Spatial Digital Predistortion for Fully-Connected Hybrid Beamforming Massive MIMO Transmitters. IEEE Trans. Circuits Syst. I Regul. Pap..

[3] Jing J., Yu C. (2020). Multibeam Digital Predistortion for Millimeter-Wave Analog Beamforming Transmitters. IEEE Microw. Wirel. Components Lett..

[4] Yu C., Jing J., Shao H., Jiang Z.H., Yan P., Zhu X.W., Hong W., Zhu A. (2019). Full-Angle Digital Predistortion of 5G Millimeter-Wave Massive MIMO Transmitters. IEEE Trans. Microw. Theory Tech..

[5] Brihuega A., Anttila L., Valkama M. (2023). Beam-Level Frequency-Domain Digital Predistortion for OFDM Massive MIMO Transmitters. IEEE Trans. Microw. Theory Tech..

[6] Braithwaite R.N. (2020). Amplifier Nonlinearities in an Antenna Array During Spatially-Multiplexed Transmissions. Proceedings of the IEEE Radio and Wireless Symposium.

[7] Bassam S.A., Helaoui M.H., Ghannouchi F.M. (2011). 2-D digital predistortion (2-D-DPD) architecture for concurrent dual-band transmitters. IEEE Trans. Microw. Theory Tech..

[8] Liu Y.J., Chen W., Zhou J., Zhou B.H., Ghannouchi F.M. (2013). Digital predistortion for concurrent dual-band transmitters using 2-D modified memory polynomials. IEEE Trans. Microw. Theory Tech..

[9] Eriksson T., Fager C. (2014). Digital predistortion of concurrent multiband communication systems. Proceedings of the 2014 IEEE International Conference on Acoustics, Speech and Signal Processing (ICASSP).

[10] Younes M., Kwan A., Rawat M., Ghannouchi F.M. (2013). Three-Dimensional digital predistorter for concurrent tri-band power amplifier linearization. Proceedings of the IEEE MTT-S International Microwave Symposium Digest (MTT).

[11] Yu C., Xia J., Zhu X.W., Zhu A. (2015). Single-Model Single-Feedback Digital Predistortion for Concurrent Multi-Band Wireless Transmitters. IEEE Trans. Microw. Theory Tech..

[12] Kolomvakis N., Bavand M., Bahceci I., Gustavsson U. (2022). A Distortion Nullforming Precoder in Massive MIMO Systems with Nonlinear Hardware. IEEE Wirel. Commun. Lett..

[13] Mollen C., Gustavsson U., Eriksson T., Larsson E.G. (2018). Spatial characteristics of distortion radiated from antenna arrays with transceiver nonlinearities. IEEE Trans. Wirel. Commun..

[14] Jing J., Shao H., Yan P., Jiang Z.H., Yu C. Digital Predistortion of Millimeter-Wave Multi-beam Transmitters with Digital Beam-forming Network. Proceedings of the 2019 IEEE MTT-S International Wireless Symposium (IWS).

[15] Molina A., Rajamani K., Azadet K. (2017). Concurrent Dual-Band Digital Predistortion Using 2-D Lookup Tables with Bilinear Interpolation and Extrapolation: Direct Least Squares Coefficient Adaptation. IEEE Trans. Microw. Theory Tech..

[16] Zhang Y., Chen Q., Peng H., Chen W., Feng Z., Ghannouchi F.M. (2026). Stream-Level Digital Predistortion for Extremely Large-Scale MIMO Transmitters. IEEE Trans. Microw. Theory Tech..

[17] Wang J., Gao K., Liu X., Zhang Y., Chen W., Feng H., Feng Z. (2024). Digital Predistortion Based on Clustering for Fully-Connected Hybrid Beamforming MIMO Transmitters with Dynamic Beam Steering. Proceedings of the 2024 IEEE MTT-S International Wireless Symposium (IWS).

[18] Wang S., Cao W., Hou R., Eriksson T. (2022). A Digital Predistortion for Concurrent Dual-Band Power Amplifier Linearization Based on Periodically Nonuniform Sampling Theory. IEEE Trans. Microw. Theory Tech..

[19] Zhou Y., Ng T.S. (2008). MIMO-OFCDM systems with joint iterative detection and optimal power allocation. IEEE Trans. Wirel. Commun..

[20] Sun B., Zhou Y., Yuan J., Shi J. (2020). Interference Cancellation Based Channel Estimation for Massive MIMO Systems with Time Shifted Pilots. IEEE Trans. Wirel. Commun..

[21] Ziao Q., Haifan Y. (2025). A review of codebooks for CSI feedback in 5G new radio and beyond. China Commun..

[22] Ning B., Yin H., Liu S., Deng H., Yang S., Zhang Y., Mei W., Gesbert D., Park J., Heath R.W. (2026). Precoding Matrix Indicator in the 5G NR Protocol: A Tutorial on 3GPP Beamforming Codebooks. IEEE Commun. Surv. Tutor..

[23] Tan F., Wu P., Wu Y.C., Xia M. (2021). Cooperative Beamforming for Wireless Fronthaul and Access Links in Ultra-Dense C-RANs with SWIPT: A First-Order Approach. IEEE J. Sel. Top. Signal Process..

[24] Li Y., Xia M., Wu Y.C. (2018). First-Order Algorithm for Content-Centric Sparse Multicast Beamforming in Large-Scale C-RAN. IEEE Trans. Wirel. Commun..

[25] Saleh A. (1981). Frequency-Independent and Frequency-Dependent Nonlinear Models of TWT Amplifiers. IEEE Trans. Commun..

[26] Chani-Cahuana J., Landin P.N., Fager C., Eriksson T. (2016). Iterative Learning Control for RF Power Amplifier Linearization. IEEE Trans. Microw. Theory Tech..

[27] Tehrani A.S., Cao H., Afsardoost S., Eriksson T., Isaksson M., Fager C. (2010). A Comparative analysis of the complexity/accuracy tradeoff in power amplifier behavioral models. IEEE Trans. Microw. Theory Tech..

[28] Blahut R.E. (2010). Fast Algorithms for Signal Processing.

[29] Ding L., Yang Z., Gandhi H. (2012). Concurrent dual-band digital predistortion. Proceedings of the 2012 IEEE/MTT-S International Microwave Symposium Digest.

